# Supplementary material for: Content-rich biological network constructed by mining PubMed abstracts
Source: BMC Bioinformatics. 2004 Oct 8;5:147. doi: 10.1186/1471-2105-5-147 (PMC528731; doi:10.1186/1471-2105-5-147)
Supplement: Additional File 2 — The original results of the above study (non-essential files are deleted to keep the file size under the limit set by BMC bioinformatics). [file 1471-2105-5-147-S2.bz2 › chilibotAdditionalFile2/dip20/312ID11278571E1364/html/ZIPA_FTSZ.html]

 


 **ZIPA** and **FTSZ** 
  
Found 24 abstracts in PubMed, retrieved 20.  
 

 What does Google say? 
 PDF only 
| .edu only 

---

**Interactive relationship** (e.g. stimulation, inhibition, etc)

**Neutral relationship**- Recruitment of  **ZipA**  to the division site by interaction with  **FtsZ** .  Ref: 10209756 Mol Microbiol, 1999
- **FtsZ**   **ZipA**  interaction represents an ancient prototype of the protein protein interaction that enables MAPs to suppress microtubule catastrophe and or to promote rescue.  Ref: 10228152 EMBO J, 1999
- **ZipA**  induced bundling of  **FtsZ**  polymers mediated by an interaction between C terminal domains.  Ref: 10960100 J Bacteriol, 2000
- In vitro, wild type  **FtsZ**  interacted with  **ZipA**  with a high affinity in an enzyme linked immunosorbent assay.  Ref: 11278571 J Biol Chem, 2001
- the  **ZipA**   **FtsZ**  interaction is mediated by their C terminal domains.  Ref: 10880432 EMBO J, 2000
- Although a MalE  **ZipA**  fusion binds to  **FtsZ**  filaments, it does not affect the GTPase activity or dynamics of the filaments.  Ref: 10209756 Mol Microbiol, 1999
- It is postulated that the MAP Tau homologous motifs in  **ZipA**  mediate its binding to  **FtsZ** ,.  Ref: 10228152 EMBO J, 1999
- A model is proposed here for how membrane bound  **ZipA**  can reach around the  **FtsZ**  protofilament to bind the carboxy terminal peptide, which faces away from the membrane.  Ref: 11163134 Curr Opin Cell Biol, 2001
- The septal ring in Escherichia coli consists of at least nine essential gene products whose order of assembly resembles a mostly linear dependency pathway FtsA and  **ZipA**  directly bind  **FtsZ**  polymers at the prospective division site, followed by the sequential addition of FtsK, FtsQ, FtsL, FtsW, FtsI, and FtsN.  Ref: 11948172 J Bacteriol, 2002
- I demonstrate that purified  **ZipA**  promotes and stabilizes protofilament assembly of both  **FtsZ**  and FtsZ84 in vitro and cosediments with the protofilaments.  Ref: 10228152 EMBO J, 1999
- In the complex, the 17 residue  **FtsZ**  fragment occupies this entire cavity of  **ZipA**  and binds as an extended beta strand followed by alpha helix.  Ref: 10880432 EMBO J, 2000
- Recent data indicate that  **ZipA**  is involved in the assembly of the ring by linking  **FtsZ**  to the cytoplasmic membrane.  Ref: 10880432 EMBO J, 2000
- Two peptide protein interactions are known in this process FtsA and  **ZipA**  bind the C terminus of  **FtsZ** , the bacterial tubulin homologue, which is the first septal component to appear at the septum.  Ref: 11254984 Biochimie, 2001
- The  **FtsZ**  binding FZB domain of  **ZipA**  is significantly larger and encompasses the C terminal 143 residues of  **ZipA** .  Ref: 10960100 J Bacteriol, 2000
- An alanine scanning mutagenesis analysis of the  **FtsZ**  fragment was also performed, which shows that only a small cluster of the buried  **FtsZ**  side chains is critical in binding to  **ZipA** .  Ref: 10880432 EMBO J, 2000
- The results show that  **ZipA**  binds to residues confined to the 20 C terminal amino acids of  **FtsZ** .  Ref: 10960100 J Bacteriol, 2000
- The interaction between  **FtsZ**  and  **ZipA**  occurs through their carboxy terminal domains.  Ref: 10209756 Mol Microbiol, 1999
- In Escherichia coli,  **FtsZ** , a homologue of eukaryotic tubulins, and  **ZipA** , a membrane anchored protein that binds to  **FtsZ** , are two essential components of the septal ring structure that mediates cell division.  Ref: 10880432 EMBO J, 2000
- FtsA and  **ZipA**  localized into a division ring following the same pattern and time course as  **FtsZ** .  Ref: 12754232 J Bacteriol, 2003
- Significantly, we find that the FZB domain of  **ZipA**  is also required and sufficient to induce dramatic bundling of  **FtsZ**  protofilaments in vitro.  Ref: 10960100 J Bacteriol, 2000
- **ZipA** , a membrane protein involved in cell division that colocalizes with  **FtsZ** , was equally dynamic.  Ref: 11854462 Proc Natl Acad Sci U S A, 2002
- **ZipA**  is a MAP Tau homolog and is essential for structural integrity of the cytokinetic  **FtsZ**  ring during bacterial cell division.  Ref: 10228152 EMBO J, 1999
- We have used polymerase chain reaction mediated random mutagenesis in the yeast two hybrid system to analyze this interaction and have identified residues within a highly conserved sequence at the C terminus of  **FtsZ**  as the  **ZipA**  binding site.  Ref: 11278571 J Biol Chem, 2001
- Characterization of  **FtsZ**  residues essential for the interactions with  **ZipA**  and with FtsA.  Ref: 11278571 J Biol Chem, 2001
- Genetic analysis of the Escherichia coli  **FtsZ** . **ZipA**  interaction in the yeast two hybrid system.  Ref: 11278571 J Biol Chem, 2001
- The cell division protein  **ZipA**   [ **ZIPA** ]  has an N terminal transmembrane domain and a C terminal globular domain that binds  **FtsZ** .  Ref: 12107152 J Bacteriol, 2002
- In contrast,  **ZipA**  was capable of binding to all four stable point mutants in the  **FtsZ**  C terminal core but not to the 12 amino acid deletion.  Ref: 10601211 J Bacteriol, 1999
- A C terminal peptide from  **FtsZ**  has been shown to bind  **ZipA**  185 328 in a hydrophobic channel formed by the beta sheet providing insight into the  **ZipA**   **FtsZ**  interaction.  Ref: 10924108 Biochemistry, 2000
- In Escherichia coli,  **FtsZ**  is required for the recruitment of the essential cell division proteins FtsA and  **ZipA**  to the septal ring.  Ref: 10601211 J Bacteriol, 1999
- Structural evidence that the P Q domain of  **ZipA**  is an unstructured, flexible tether between the membrane and the C terminal  **FtsZ**  binding domain.  Ref: 12107152 J Bacteriol, 2002
- These results are consistent with  **ZipA**  acting after Z ring formation, possibly to link the membrane to  **FtsZ**  filaments during invagination of the septum.  Ref: 10209756 Mol Microbiol, 1999
- **ZipA** , an essential component of cell division in Escherichia coli, interacts with the  **FtsZ**  protein at the midcell in one of the initial steps of septum formation.  Ref: 10924108 Biochemistry, 2000
- We have shown that a fusion, which encodes the last 70 residues of  **FtsZ**  in the two hybrid system, is sufficient for the interaction with FtsA and  **ZipA** .  Ref: 11278571 J Biol Chem, 2001
- The bacterial cell division protein  **ZipA**   [ **ZIPA** ]  and its interaction with an  **FtsZ**  fragment revealed by X ray crystallography.  Ref: 10880432 EMBO J, 2000
- **ZipA**  and FtsA are essential division proteins in Escherichia coli that are recruited to the division site by interaction with  **FtsZ** .  Ref: 11847116 EMBO J, 2002
- Furthermore,  **ZipA**  organizes  **FtsZ**  protofilaments into arrays of long bundles or sheets that probably represent the physiological organization of the  **FtsZ**  ring in bacterial cells.  Ref: 10228152 EMBO J, 1999
- **ZipA** , a membrane tethered division protein, binds to a highly conserved short peptide on the carboxyl terminus of  **FtsZ** .  Ref: 11163134 Curr Opin Cell Biol, 2001
- **ZipA**  is an inner membrane protein which is recruited to the nascent septal ring at a very early stage through a direct interaction with  **FtsZ** .  Ref: 10960100 J Bacteriol, 2000
- An unexpected similarity between the  **ZipA**  185 328 fold and the split beta alpha beta fold observed in many RNA binding proteins may further our understanding of the critical  **ZipA**   **FtsZ**  interaction.  Ref: 10924108 Biochemistry, 2000

**Non-interactive relationship** (e.g. studied together, co-existance, homology, etc.)

- Concentration and Assembly of the Division Ring Proteins  **FtsZ** , FtsA, and  **ZipA**  during the Escherichia coli Cell Cycle.  Ref: 12754232 J Bacteriol, 2003
- The recruitment of  **ZipA**  to the septum by  **FtsZ**  is an early, essential step in cell division in Escherichia coli.  Ref: 11278571 J Biol Chem, 2001
- These localization results support the model that E. coli cell division proteins assemble sequentially as a multimeric complex at the division site first  **FtsZ** , then FtsA and  **ZipA**  independently of each other, followed successively by FtsK, FtsQ, FtsL, FtsW, FtsI and FtsN.  Ref: 11703663 Mol Microbiol, 2001
- Consistent with the notion that the ability to bind and bundle  **FtsZ**  polymers is essential to the function of  **ZipA** , we find that  **ZipA**  derivatives lacking an intact FZB domain fail to support cell division in cells depleted for the native protein.  Ref: 10960100 J Bacteriol, 2000
- The concentration of the cell division proteins  **FtsZ** , FtsA, and  **ZipA**  and their assembly into a division ring during the Escherichia coli B r K cell cycle have been measured in synchronous cultures obtained by the membrane elution technique.  Ref: 12754232 J Bacteriol, 2003
- Mutations surrounding Asp 373 differentially affected the interactions of  **FtsZ**  with  **ZipA**  and FtsA, indicating that these proteins bind the C terminus of  **FtsZ**  differently.  Ref: 11278571 J Biol Chem, 2001
- The  **FtsZ**  protofilament and attachment of  **ZipA**  structural constraints on the  **FtsZ**  power stroke.  Ref: 11163134 Curr Opin Cell Biol, 2001
- In a reverse approach, we use fusions of green fluorescent protein GFP to  **FtsZ** , FtsA and  **ZipA**  to show that these proteins localize to the division site in an FtsL independent fashion.  Ref: 10027987 Mol Microbiol, 1999
- **ZipA**  and FtsA are recruited independently to the  **FtsZ**  cytokinetic ring Z ring and are essential for cell division of Escherichia coli.  Ref: 12634424 Proc Natl Acad Sci U S A, 2003
- MinCD prevented the localization of both  **FtsZ**  GFP and  **ZipA**  GFP, consistent with it preventing Z ring assembly.  Ref: 11673433 J Bacteriol, 2001
- **FtsZ**  and  **ZipA**  are essential components of the septal ring apparatus, which mediates cell division in Escherichia coli.  Ref: 10960100 J Bacteriol, 2000
- In Escherichia coli, at least nine essential gene products participate directly in septum formation FtsA, FtsI, FtsL, FtsK, FtsN, FtsQ, FtsW,  **FtsZ**  and  **ZipA** .  Ref: 11703663 Mol Microbiol, 2001
- In this paper, size exclusion chromatography was used to study the effect of the SulA and MinCD division inhibitors on the oligomerization state of endogenous  **FtsZ**  in cytoplasmic extracts, and immunofluorescence microscopy was used to determine the effect of SulA and MinCD on the formation of  **FtsZ** , FtsA and  **ZipA**  rings at potential division sites.  Ref: 10931335 Mol Microbiol, 2000
- In contrast, the MinCD division inhibitor did not prevent the oligomerization of  **FtsZ**  in the cell extracts or the formation of  **FtsZ**  and  **ZipA**  ring structures in vivo.  Ref: 10931335 Mol Microbiol, 2000
- We present the X ray crystal structures of the C terminal  **FtsZ**  binding domain of  **ZipA**  and a complex between this domain and a C terminal fragment of  **FtsZ** .  Ref: 10880432 EMBO J, 2000
- Because another proposed function of  **FtsZ**  is the recruitment of FtsA and  **ZipA** , we tested whether the C terminal core domain was important for interactions with these proteins.  Ref: 10601211 J Bacteriol, 1999
- **ZipA**  requires  **FtsZ** , but not FtsA or FtsI, to be localized, indicating that it is recruited by  **FtsZ** .  Ref: 10209756 Mol Microbiol, 1999
- Consistent with this, we found that an  **FtsZ**  mutant unable to interact with both  **ZipA**  and FtsA was unable to assemble into Z rings.  Ref: 11847116 EMBO J, 2002
- The results imply that association of both FtsA and  **ZipA**  with  **FtsZ**  polymers is needed for further maturation of the nascent organelle.  Ref: 11948172 J Bacteriol, 2002
